# Supplementary material for: Research on boundary control of vehicle-mounted flexible manipulator based on partial differential equations
Source: PLoS One. 2025 Jan 7;20(1):e0317012. doi: 10.1371/journal.pone.0317012 (PMC11706493; doi:10.1371/journal.pone.0317012)
Supplement: S1 File — (PDF) [file pone.0317012.s001.pdf]

```
close all;
clear all;
nx = 10;
nt = 80000;   % 保持时间步数不变
tmax = 40;   % 保持时间不变
L = 1.0;   % 新的长度
% 计算空间步长和时间步长
dx = L / (nx - 1);   % 更新空间步长以适应新的长度 L
T = tmax / (nt - 1);   % 时间步长保持不变
% 创建数组以保存数据用于导出
t = linspace(0, tmax, nt);
x = linspace(0, L, nx);   % 根据新的长度 L 更新
%Parameters
EI=2;m=6.78;rho=0.2211;Ih=0.0139;
thd=0.5;dthd=0;ddthd=0;
kp=30;kd=50;k=10;
F_1=0;
%Define viriables and Initial condition:
y=zeros(nx,nt);     %elastic deflectgion
th_2=0;th_1=0;
dth_1=0;
for j=1:nt
    th(j)=0;     %joint angle
end
for j=3:nt      %Begin
e(j)=th_1-thd;
de(j)=dth_1-dthd;
```

```
%th(j)
yxx0=(y(3,j-1)-2*y(2,j-1)+y(1,j-1))/dx^2;
tol(j)=-kp*e(j)-kd*de(j);     %PD control for the joint
th(j)=2*th_1-th_2+T^2/Ih*(tol(j)+EI*yxx0);     %(A1)
dth(j)=(th(j)-th_1)/T;
ddth(j)=(th(j)-2*th_1+th_2)/T^2;
%get y(i,j),i=1,2, Boundary condition (A2)
y(1,:)=0;       %y(0,t)=0, i=1
y(2,:)=0;       %y(1,t)=0, i=2
%get y(i,j),i=3:nx-2
for i=3:nx-2
    yxxxx=(y(i+2,j-1)-4*y(i+1,j-1)+6*y(i,j-1)-4*y(i-1,j-1)+y(i-2,j-1))/dx^4;
    y(i,j)=T^2*(-i*dx*ddth(j)-EI*yxxxx/rho)+2*y(i,j-1)-y(i,j-2);   %i*dx=x, (A3)
    dy(i,j-1)=(y(i,j-1)-y(i,j-2))/T;
end
%get y(nx-1,j),i=nx-1
yxxxx(nx-1,j-1)=(-2*y(nx,j-1)+5*y(nx-1,j-1)-4*y(nx-2,j-1)+y(nx-3,j-1))/dx^4;
y(nx-1,j)=T^2*(-(nx-1)*dx*ddth(j)-EI*yxxxx(nx-1,j-1)/rho)+2*y(nx-1,j-1)-y(nx-1,j-2);   %(A6)
dy(nx-1,j)=(y(nx-1,j)-y(nx-1,j-1))/T;
%get y(nx,j),y=nx
yxxx_L=(-y(nx,j-1)+2*y(nx-1,j-1)-y(nx-2,j-1))/dx^3;
y(nx,j)=T^2*(-L*ddth(j)+(EI*yxxx_L+F_1)/m)+2*y(nx,j-1)-y(nx,j-2);     %(A7)
dy(nx,j)=(y(nx,j)-y(nx,j-1))/T;
%%%%%%%%%%%%%%%%%%%%%%%%%%%%%
dzL=L*dth(j)+(y(nx,j)-y(nx,j-1))/T;
```

```
F(j)=-k*dzL; %P Control for the end
F_1=F(j);
th_2=th_1;
th_1=th(j);
dth_1=dth(j);
end     %End
%To view the curve, short the points
tshort=linspace(0,tmax,nt/100);
yshort=zeros(nx,nt/100);
dyshort=zeros(nx,nt/100);
for j=1:nt/100
        for i=1:nx
            yshort(i,j)=y(i,j*100);     %Using true y(i,j)
            dyshort(i,j)=dy(i,j*100);     %Using true dy(i,j)
        end
end
thd1 =[];
for i = 1:80000
      thd1(i) = thd;
end
      %%%%%%%%%%%%%%%%%%%%%%%%%%
figure(1);
subplot(211);
plot(t,thd1,'r',t,th,'b--','linewidth',3);
title('Joint angle tracking');
xlabel('time(s)');ylabel('angle tracking');
```

```
legend('thd','th');
subplot(212);
plot(t,dth,'k','linewidth',3);
xlabel('Time (s)');ylabel('Angle speed response (rad/s)');
legend('dth');

figure(2);
surf(tshort,x,yshort);
title('Elastic deflection of the flexible arms');
xlabel('time(s)'); ylabel('x');zlabel('deflection,y(x,t)');

figure(3);
surf(tshort,x,dyshort);
xlabel('Time (s)'); ylabel('x');zlabel('Deflection rate, dy(x,t) (m/s)');

figure(4);
subplot(211);
for j=1:nt/100
    yshortL(j)=y(nx,j*100);
end
plot(tshort,yshortL,'r','linewidth',3);
xlabel('Time (s)');ylabel('y(L,t)');
subplot(212);
for j=1:nt/100
    yshort1(j)=y(nx/2,j*100);
end
plot(tshort,yshort1,'r','linewidth',3);
```

```
xlabel('Time (s)');ylabel('y(x,t) at half of L');


figure(5);
plot(t,tol,'r','linewidth',3);
xlabel('Time (s)');ylabel('control input,tol');


figure(6);
plot(t,F,'r','linewidth',3);
xlabel('Time (s)');ylabel('control input,F');
```
