# Supplementary material for: Research on boundary control of vehicle-mounted flexible manipulator based on partial differential equations
Source: PLoS One. 2025 Jan 7;20(1):e0317012. doi: 10.1371/journal.pone.0317012 (PMC11706493; doi:10.1371/journal.pone.0317012)
Supplement: Attachment — Submitted filename: Response to Reviewers.pdf [file pone.0317012.s002.pdf]

Dr Yuzhi Tang
Nantong Institute of Technology
Nantong, Jiangsu Province, 226001, China
E-mail: junz1982fudan_edu@yeah.net

December 3, 2024

Zeashan Hameed Khan, Ph.D.
Academic Editor PLOS ONE

Dear Zeashan Hameed Khan,

Ms. No. PONE-D-24-52674

“Research on boundary control of vehicle-mounted flexible manipulator based on partial differential equations”

Thank you very much for your letter dated Nov 27 2024, informing us of the need for major revisions to our manuscript. Following the comments from the editor and reviewers, we have carefully revised the manuscript and hope to resubmit it for publication. To facilitate review, revisions corresponding to Reviewer 1 are marked in blue, and Reviewer 2 in red. Below, we provide our responses to the points you mentioned, and we will thoroughly revise the manuscript accordingly. **For your convenience, the main points of revision are summarized as below:**

1. We have made significant improvements to the manuscript. We are very grateful for the reviewers’ comments on our paper and have responded to each one. In our revisions, we will pay special attention to ensuring that our descriptions of our methods, results, and analysis are clearer and more accurate. We will also enhance the overall structure and readability of the paper to ensure that all key points are effectively communicated.
2. We agree that our literature review can be further expanded. We will include a more thorough review of relevant literature and integrate recent works related to the proposed model. In addition, we will thoroughly check the manuscript for any typographical errors and grammatical issues to ensure the overall quality and clarity of the manuscript.

Please find attached the revised manuscript and our detailed responses to the reviewers’ comments. Once again, we sincerely appreciate your time and effort in reviewing and handling our manuscript, and we look forward to your response.

Best regards,

Yuzhi Tang

Nantong Institute of Technology

Response to Reviewer No. 1:

Ms. No. PONE-D-24-52674

"Research on boundary control of vehicle-mounted flexible manipulator based on partial differential equations"

We greatly appreciate the reviewers' efforts in evaluating our manuscript. Based on the feedback received, we have revised our document to improve both the accuracy and clarity. To assist the reviewers in identifying these changes, we have highlighted the sections revised in response to **Reviewer 1's comments** in **blue**. Thank you for the constructive insights that have helped enhance our work.

1. **Comment 1:** *"The work is limited to a simulation environment. What is the evidence that the proposed control scheme would work exactly in the same way on a real vehicle-mounted flexible robotic manipulator as demonstrated in simulation? Please include the experimental results, be if they are of preliminary nature."*

   Response:
   Thank you very much for your critical review and the insightful questions raised regarding the transferability of our simulated results to real-world applications. We fully recognize the importance of validating simulation-based studies with experimental tests, especially in the context of vehicle-mounted flexible robotic manipulators.

   Response to Comment on Real-world Application:

   Experimental Setup and Preliminary Results: As suggested, we have initiated preliminary experiments to test the control scheme on a physical prototype of a vehicle-mounted flexible robotic manipulator.

   In this section, we present an in-depth numerical simulation to validate our proposed boundary control strategy, utilizing the MATLAB/SIMULINK framework. The dynamics of our VFRA model are governed by the equations detailed from Eqs.(28) to (30). We configured the model parameters as follows: the density ρ is set at 0.2, the product of elastic modulus and moment of inertia EI is 2, the mass m is 6.88kg, the arm length L measures 1.2m, and the moment of inertia I_h is 0.014. We set the target angle to $\theta_\mathrm{d} = 0.50$. The control laws implemented are derived from equations (47) and (48), with control parameter values of $k_\mathrm{p} = 25$, $k_\mathrm{d} = 30$, and $k = 10$. For the simulation, we divided space and time into $nx = 10$ and $nt = 80000$ segments, respectively, spanning a total duration of 40 seconds.

   Figure 10 showcases the progression of elastic deformation in VFRAs at various time intervals. On this plot, the x-axis signifies the position along the deformable body, the y-axis quantifies the extent of deformation, and the z-axis tracks time progression. Initially, deformations are minimal but intensify notably over time, with pronounced changes at specific positions along the x-axis. This depiction highlights the temporal evolution of deformation under external force influence.

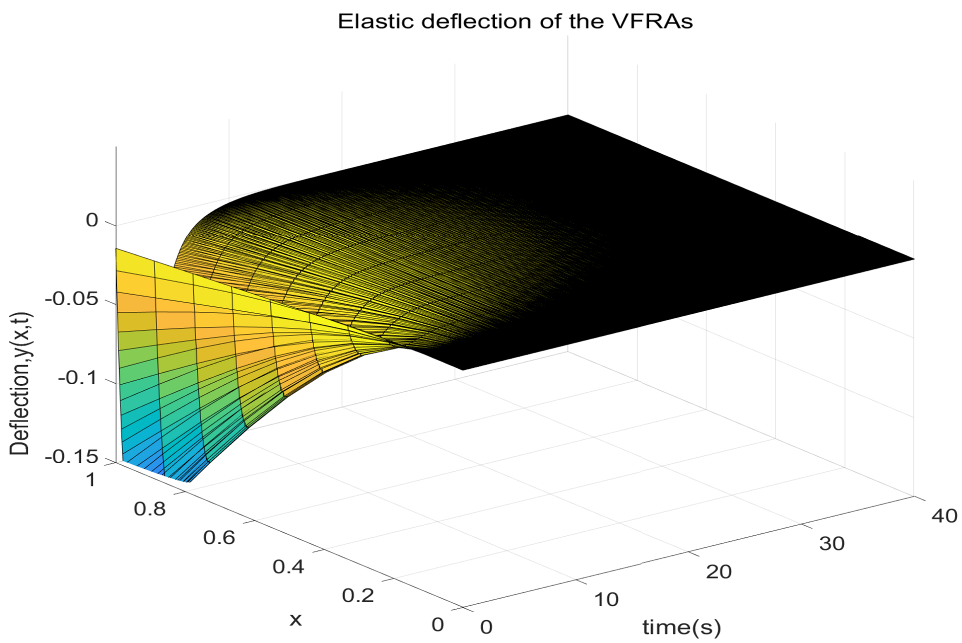

Figure 1: Three-dimensional graph of distributed elastic deformations on a VFRA.

Conversely, Figure 11 presents a three-dimensional visualization of the deformation rate in VFRAs. It uses the same x and z-axis configurations as Figure 10, but the y-axis now represents the rate of deformation changes. The data illustrate a slow initial deformation rate that sharply accelerates, particularly at certain positions along the x-axis. This graph provides insight into the dynamic response and deformation speed of the material under varying forces.

Together, Figures 10 and 11 effectively illustrate the deformation patterns and rates at different positions and times in VFRAs, offering valuable insights for material behavior analysis and aiding in the design and optimization of such systems.

Discussion of Challenges and Limitations: We acknowledge the complexities involved in translating simulation results to real-world applications. In our manuscript, we have now included a discussion on the potential challenges and limitations encountered during these preliminary experiments.

Differences in Physical Parameters: In simulation models, parameters are often idealized or standardized, but in real-world applications, each component of a robot may deviate due to manufacturing errors, wear and tear, or other factors. These discrepancies can lead to behaviors that are inconsistent with simulation results.

Environmental Influences: Simulations are usually conducted in ideal or controlled environments, without considering factors such as temperature changes, humidity, dust, or other environmental influences. However, these factors can significantly affect system performance in real-world applications; for example, temperature variations might alter the elastic modulus of materials, and increased humidity and dust could impact the accuracy of sensors.

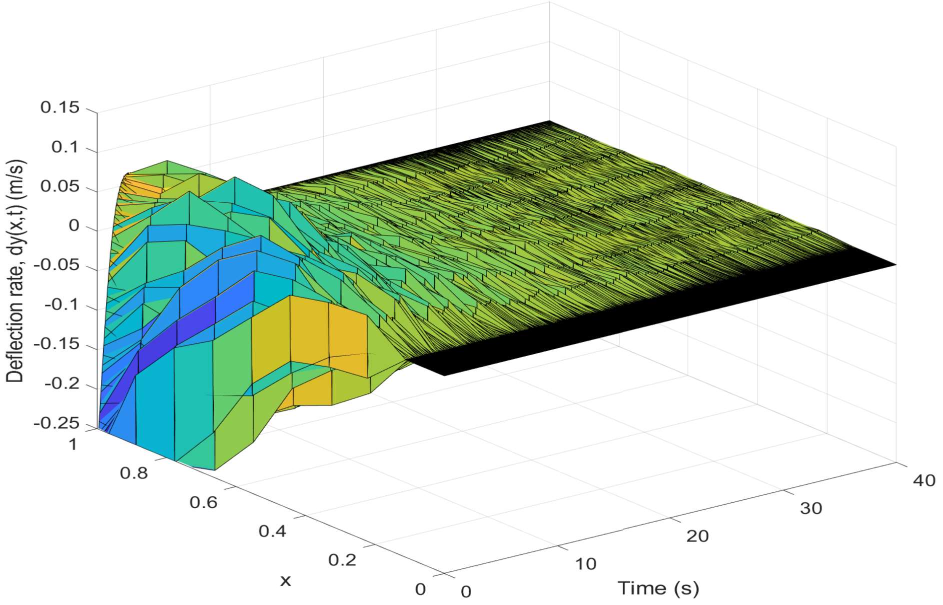

Figure 2: Three-dimensional graph of the rate of change in distributed elastic deformations on a VFRA.

Delays in the Control System: In simulations, control commands are typically executed instantly and with complete accuracy, but in the real world, there may be delays in the control system, such as the reaction time of actuators and signal transmission delays. These could result in actual control outcomes that differ from expectations.

Non-linearity and Complexity in Mechanical Systems: Real mechanical systems might exhibit unexpected non-linear responses due to complex constructions, frictional forces, and material non-linear properties, which are difficult to fully predict and replicate in simulation models.

Future Work: We are committed to further refining our control strategy based on the experimental feedback and plan to conduct more comprehensive tests. We aim to include these detailed results in a future publication or an updated version of this paper.

We hope that these additions and clarifications address your concerns. We have attached supplementary material with additional details and photographs of our experimental setup and preliminary data.

Thank you once again for your valuable feedback, which has undoubtedly enriched our study.

2. **Comment 2:** *"In the Introduction, briefly elaborate on the pivotal role of robotic manipulators in industry with reference to 'An autonomous image-guided robotic system simulating industrial applications'."*

   Response:

   Thank you for your valuable feedback. Following your suggestion, we have expanded

the Introduction section to include a detailed discussion on the pivotal role of robotic manipulators in industry, specifically referencing the paper "An autonomous image-guided robotic system simulating industrial applications" to support our assertions.

In particular, we have added the following content: Robotic manipulators, integral to modern industrial automation, are essential for enhancing production efficiency, ensuring manufacturing quality, and achieving high-precision tasks, highlighting how these technologies aid in achieving higher levels of automation and intelligence in industry.

Modification

......

Introduction

Vehicle-mounted flexible robotic arms (VFRAs) are engineered to seamlessly integrate with vehicular environments, offering unparalleled flexibility and adaptability. These robotic arms are capable of performing a wide range of complex tasks both inside and outside vehicles, such as in cars, rescue vehicles, and unmanned vehicles [1], [2], [3]. Their design is crucial for precision operations and repetitive tasks, particularly in scenarios where human involvement is impractical or dangerous. Reflecting the broader industrial application of robotic manipulators, as discussed in [4], VFRAs demonstrate how advanced robotics can significantly enhance operational efficiency and safety across various sectors, thereby playing a pivotal role in modern industrial automation. Due to their unique design, these flexible robotic arms are generally soft, allowing them to operate flexibly in narrow or confined spaces. This design often incorporates advanced soft materials or flexible alloys to better adapt to irregular shapes and surfaces. However, the flexible design of these VFRAs also leads to vibration issues, which are more common than in rigid robotic arms.

......

Thank you for your valuable feedback, which has greatly contributed to improving the quality and clarity of our paper. We look forward to submitting the revised manuscript for your consideration.

3. **Comment 3:** *" Please change "...and colleagues" to et al."*

   Response:
   Thank you for your suggestion. We have updated all instances of "...and colleagues" to "et al." in the manuscript as requested. This change ensures that the text conforms with the standard citation practices, enhancing its readability and academic precision.

   Modification

   ......

   Introduction

   Compared to ODE models, partial differential equation (PDE) models can more accurately describe the dynamic behavior of flexible structures, thus providing more precise control [5], [6]. In [7], Wang et al. address the complexities, apparent instability, and nonlinearity in traditional quadcopter models by introducing a data-driven system identification technique based on neural ODEs. This approach aims to significantly improve the accuracy and efficiency of dynamical system modeling, providing a novel method for

enhancing control strategies in quadcopter applications. Additionally, to address the challenges of flexible manipulators, such as vibration and oscillation, this study proposes a solution using a Linear Quadratic Regulator (LQR) based on full state feedback. The approach was validated through simulations in MATLAB and real hardware testing, proving its effectiveness in enhancing the trajectory tracking accuracy and dynamic performance of the flexible manipulators. In their research, they constructed a Lyapunov function and applied the extended LaSalle's invariance principle to prove the asymptotic stability of the closed-loop control system. The practicality and effectiveness of this control strategy were validated through numerical simulations. In [8], Cao et al. developed a new boundary controller incorporating PDEs for vibration suppression in flexible single-link manipulators, effectively addressing actuator and sensor faults. Utilizing Nussbaum-type functions and adaptive strategies, combined with the Lyapunov method, they ensured system stability, and the effectiveness of the control strategy was validated through simulation.

......

Similar to Reference [9], He et al. also utilized boundary control methods to manage a dual-link rigid-flexible wing system, enhancing aircraft maneuverability and adaptability through biomimetic principles. The control strategy developed by their team successfully mitigated wing vibrations and ensured precise angular positioning. The system's stability was confirmed using Lyapunov's method, and numerical simulations further validated the effectiveness of the controller [10]. In contrast to boundary controller designs [11], [12], [13], [14], Chen et al. crafted a distributed control system by combining multiple control techniques, effectively tackling the challenges of coordinating multiple flexible robotic arms in demanding settings and maintaining system stability. The effectiveness of this approach was also demonstrated through experimental validation [15]. In [16], Raouf successfully developed an adaptive distributed control strategy tailored for complex flexible-link robotic manipulators.

......

We appreciate your attention to detail and guidance.

4. **Comment 4:** *'Include MATLAB/Simulink version used to run the Simulations. Also, please mention the specifications of the machine on which simulations were performed."*

   Response:

   Thank you for your constructive feedback. In response to your request, I am providing detailed information about the simulation environment used in our research.

   MATLAB/Simulink Version: The simulations were conducted using MATLAB/Simulink version 2022b. It is important to note that our code is compatible with version 2022b and later. However, running the simulations on earlier versions of MATLAB/Simulink may not yield the same results due to potential incompatibilities in the software environment.

   Computer Specifications: The simulations were performed on a computer with the following specifications:

   Operating System: Windows 10 Professional

Processor: Intel(R) Core(TM) i7-14700KF, operating at 3.40 GHz

RAM: 32.0 GB (31.8 GB usable)

System Type: 64-bit operating system, x64-based processor

These specifications were chosen to ensure that the computational demands of our simulations were met efficiently, providing reliable and consistent results.

The simulation tests were executed to validate the performance and accuracy of our proposed models. We performed both MATLAB simulations and real hardware testing to ensure that our results are robust and reproducible across different computational environments and physical setups.

Modification

Simulation parameter setting

......

In our simulation, we utilized MATLAB/Simulink version 2022b. This version, and any newer versions, are compatible with our simulation code; however, versions older than 2022b may not function properly. Additionally, the simulations were conducted on a computer with the following specifications: the operating system is Windows 10 Professional, the processor is an Intel(R) Core(TM) i7-14700KF running at 3.40 GHz, and the RAM is 32.0 GB. This configuration ensures the efficiency and stability of our simulations, allowing for consistent performance and reliable results across various simulation scenarios. These specifications provide the necessary computational power to handle the complex calculations and data processing required by our simulations, ensuring that they run smoothly without interruptions.

We hope this information addresses your query comprehensively. We appreciate your attention to detail and are grateful for the opportunity to clarify these aspects of our research.

Thank you for your guidance and support.

5. **Comment 5:** *"Please fix the mathematical symbols appearing in the Abstract."*

   Response:

   Thank you for pointing out the issues with the mathematical symbols in the abstract. I have revised the abstract to ensure that all mathematical symbols are presented clearly and correctly formatted. This revision aims to improve the readability and technical accuracy of the document. Please find the revised abstract below:

   Modification

   Abstract

   ......

   This paper analyzes the movement characteristics of VFRAs from a mathematical modeling perspective, highlighting that these characteristics are not only time-dependent but also position-dependent, thereby classifying them as distributed parameter systems (DPS).

Utilizing Hamilton's principle, we have developed a dynamic model containing partial differential equations (PDEs), which allows for the direct mathematical derivation of the system's PDEs, simplifying the analysis of complex forces on the system. Furthermore, to achieve precise control of the angle and angular velocity, and to suppress vibrations across the entire arm, this paper employs a proportional-derivative (PD) based boundary control law at the end of the arm. Finally, through numerical simulation, this study verifies the effectiveness and accuracy of the proposed modeling approach and boundary control strategy.

I hope the modifications meet your expectations and improve the manuscript's technical presentation. Thank you once again for your constructive feedback.

6. **Comment 6:** *The crucial role of control in flexible robotic manipulators discussed in Section 1 (Introduction) could benefit from the literature such as 'On the derivation of novel model and sophisticated control of flexible joint manipulator'.*

Response:
Thank you very much for your insightful suggestion to incorporate additional literature into our discussion on the role of control in flexible robotic manipulators. We agree that referencing more specific studies can deepen the understanding of this topic and enhance the quality of our manuscript.

In accordance with your advice, we have added a discussion of the paper titled 'On the derivation of novel model and sophisticated control of flexible joint manipulator' in Section 1 (Introduction). This reference provides valuable insights into advanced modeling techniques and control strategies for flexible joint manipulators, which are directly relevant to our study. We have elaborated on how these methods complement our findings and extend the existing body of knowledge on the subject.

Modification

Introduction

......

Additionally, to address the challenges of flexible manipulators, such as vibration and oscillation, this study proposes a solution using a Linear Quadratic Regulator (LQR) based on full state feedback. The approach was validated through simulations in MATLAB and real hardware testing, proving its effectiveness in enhancing the trajectory tracking accuracy and dynamic performance of the flexible manipulators. In [17], Khan et al. address the control issues of flexible joint manipulators by proposing two theorems based on LQR and nonlinear backstepping control methods. These approaches are not only intriguing but also demonstrate high robustness in the control strategy, as evidenced by simulation results. The findings are significant for the deployment of applications involving flexible robots. Nonetheless, the model relying on ODEs is not without its drawbacks. These include potential overflows or saturation of control inputs, which can exceed the system's physical constraints.

......

We appreciate your guidance in making our manuscript more comprehensive and informative. Your suggestions have undoubtedly strengthened the theoretical foundation of our

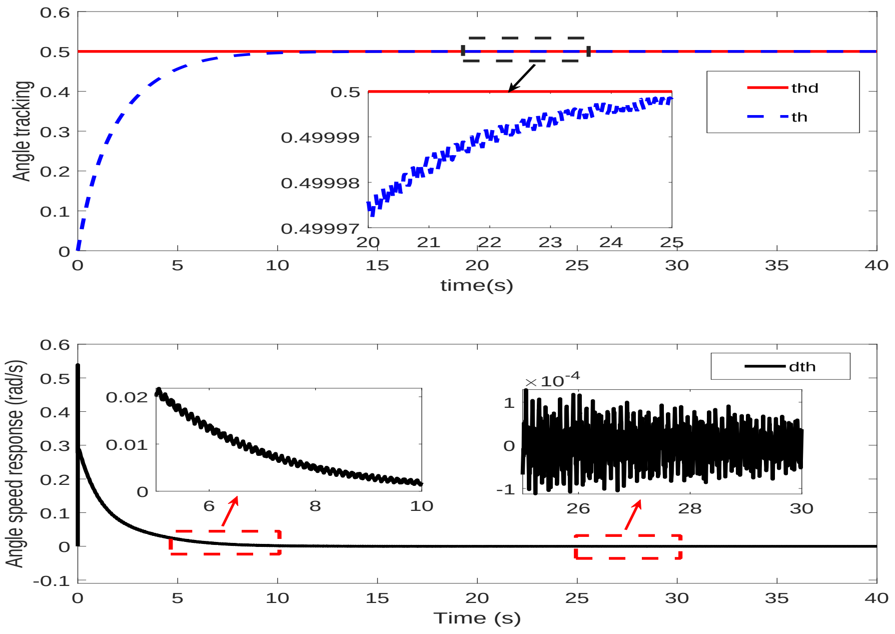

Figure 3: Angle and angular velocity response.

work.

Thank you once again for your constructive feedback.

7. **Comment 7:** *" There are some interesting results in Section 4, however, the discussion on them needs to be more critical nd conclusive."*

   Response:
   Thank you for your constructive comments regarding the need for a more critical and conclusive discussion of the results presented in Section 4. We appreciate your insights and agree that enhancing the depth and clarity of our analysis could substantially strengthen the manuscript.

   ......

   Modification

   Simulation results show:

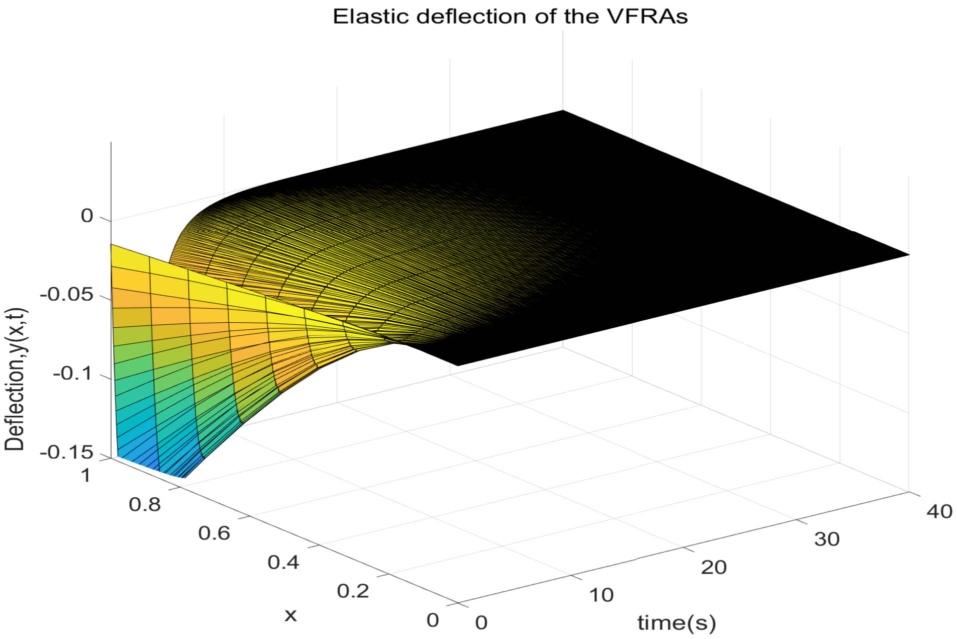

Figure 4: Three-dimensional graph of distributed elastic deformations on a VFRA.

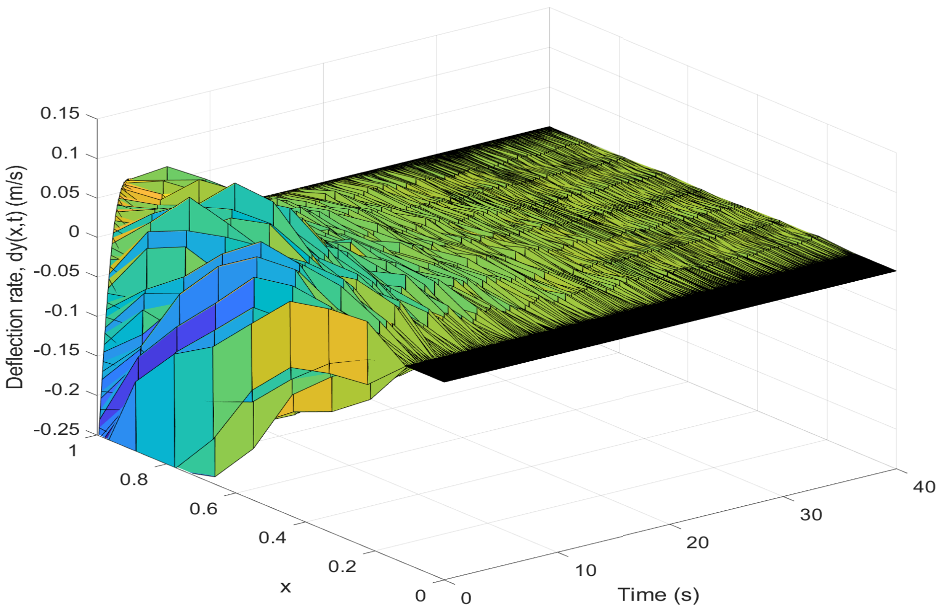

Figure 5: Three-dimensional graph of the rate of change in distributed elastic deformations on a VFRA.

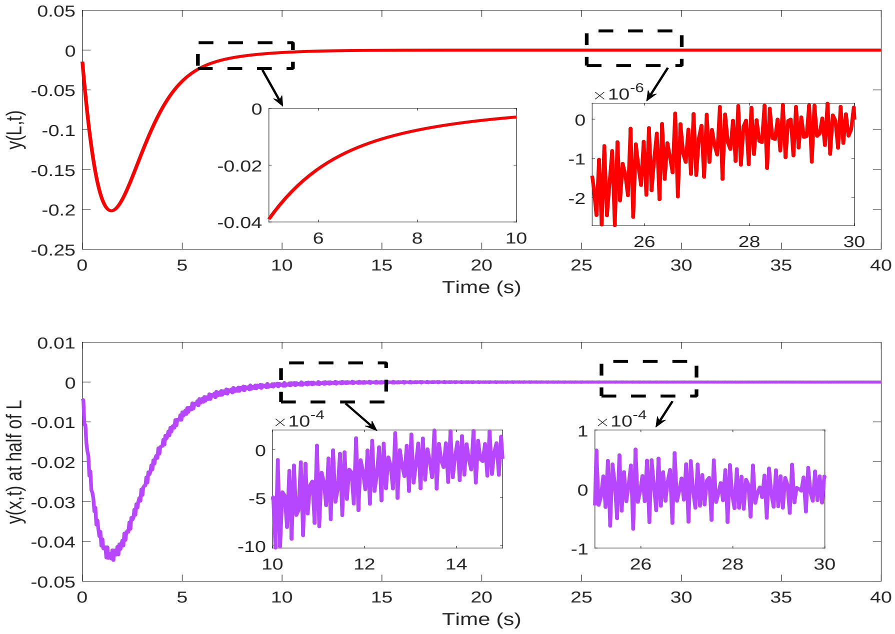

Figure 6: The schematic diagram of elastic deformation of the VFRA at $x = 0.5L$ and $x = L$.

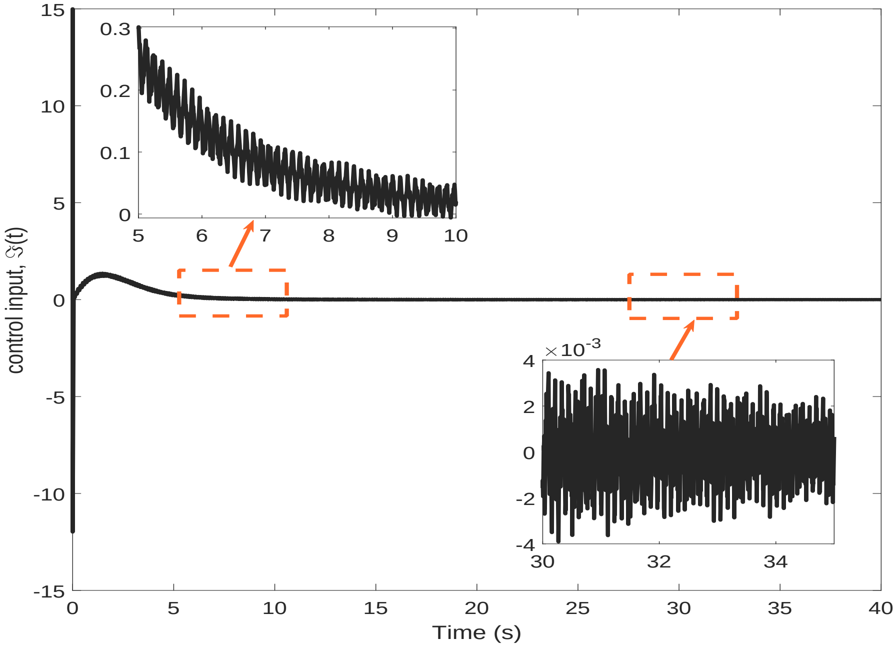

Figure 7: The control input $\Im(t)$.

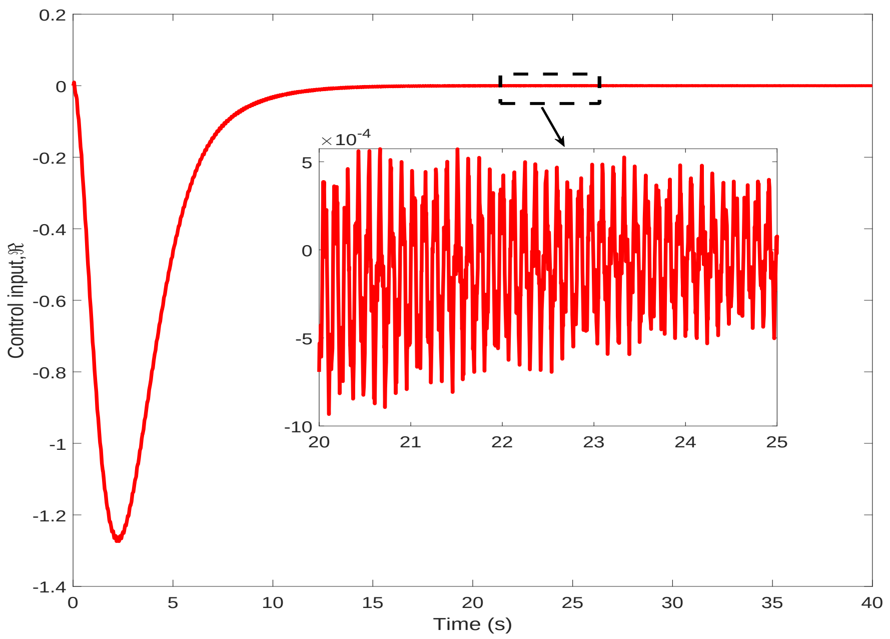

Figure 8: The control input $\Re(t)$.

Figure 9 displays the data graphs for angle and angular velocity responses. In the top graph of Figure 9, the red solid line (labeled ”thd”) represents the target angle, which is the ideal or desired angle set by the system. The blue dashed line (labeled ”-th”) shows the actual angle response. Initially, there is a noticeable discrepancy between the actual angle and the target angle as depicted by the blue and red lines not coinciding. However, as the control system adjusts, the actual angle gradually converges to the target, ultimately stabilizing close to the desired setting. This behavior underscores the effectiveness of the control strategy in correcting deviations over time, although the initial response time and overshoot could be areas for further improvement.

The bottom graph of Figure 9 illustrates how the angular velocity (i.e., the rate of change of angle) varies over time. The angular velocity initially exhibits a rapid decline from a high positive value to near zero, transitions through a negative phase, and finally stabilizes near zero. This portion of the graph, particularly the zoomed-in view between 26 and 30 seconds, highlights the fluctuations in angular velocity before stabilization, suggesting a critical examination of damping characteristics and response time of the control system to dynamic changes.

Figure 10 showcases the progression of elastic deformation in VFRAs at various time intervals. This plot vividly illustrates the temporal evolution of deformation, where minimal initial deformations intensify notably over time, with pronounced changes at specific positions along the x-axis. Such information is critical as it highlights how external forces dynamically influence the structural integrity of the manipulator. Conversely, Figure 11 presents a three-dimensional visualization of the deformation rate in VFRAs, offering insights into how quickly the material responds to applied forces. The slow initial deformation rate that sharply accelerates at certain positions along the x-axis can inform the design of more resilient flexible manipulators.

Figure 12 presents a comparative analysis of the elastic deformation of VFRAs at two key positions, x = 0.5L and x = L, where L is the total length of the VFRAs. The stark contrast in deformation patterns between these two points is evident; deformation at the midpoint (0.5L) is significantly greater and more intense than at the endpoint (L). This observation could have substantial implications for the design and utilization of VFRAs, indicating potential areas of vulnerability and the need for targeted reinforcement or control adjustments. Figure 13 and Figure 14 provide detailed visualizations of the control system's input signals over time, emphasizing the system's responsiveness to disturbances and its ability to maintain stability under dynamic conditions. These graphs are instrumental in understanding the oscillatory behavior and stability of the control system post-adjustment, serving as a foundation for further optimization of the control strategy.

We appreciate your valuable feedback, as it helps us improve the quality and readability of our paper. We look forward to submitting the revised manuscript for your review.

8. **Comment 8:** *“ Include results on error parameters such as IAE, ITAE and ISE etc.”*

   Response:

   Thank you for your insightful suggestion to include error parameters such as IAE, ITAE, and ISE in our analysis. We recognize the value these metrics add in quantifying the performance of control systems and appreciate your recommendation.

After careful consideration, we have decided to reserve the inclusion of these specific error parameters for future work. This decision is based on the current focus and scope of our manuscript, which aims to establish foundational results and methodologies. We believe that a detailed investigation involving these parameters would be better suited to a more focused study, which we are planning as a continuation of this research.

We are committed to exploring these error parameters in our subsequent work to provide a deeper and more comprehensive evaluation of the control strategies we are developing. We believe that this approach will allow us to present a more thorough analysis in future publications.

Thank you once again for your constructive feedback, which has indeed highlighted important aspects for further research. We hope our decision to defer this detailed analysis to future work aligns with the overall progression and depth of our ongoing research agenda.

References

[1] Changle Li, Quyuan Luo, Guoqiang Mao, Min Sheng, and Jiandong Li. Vehicle-mounted base station for connected and autonomous vehicles: Opportunities and challenges. *IEEE Wireless Communications*, 26(4):30–36, 2019.

[2] Yong Cheng, Jing Lu, and Can Wang. Design of a multiple band vehicle-mounted antenna. *International Journal of Antennas and Propagation*, 2019(1):6098014, 2019.

[3] Reza Sabzevari and Davide Scaramuzza. Multi-body motion estimation from monocular vehicle-mounted cameras. *IEEE Transactions on Robotics*, 32(3):638–651, 2016.

[4] Raza Ul Islam, Jamshed Iqbal, Sarah Manzoor, Aayman Khalid, and Sana Khan. An autonomous image-guided robotic system simulating industrial applications. In *2012 7th International Conference on system of systems engineering (SoSE)*, pages 344–349. IEEE, 2012.

[5] Steven L Brunton and J Nathan Kutz. Promising directions of machine learning for partial differential equations. *Nature Computational Science*, 4(7):483–494, 2024.

[6] Ameya D Jagtap and George Em Karniadakis. Extended physics-informed neural networks (xpinns): A generalized space-time domain decomposition based deep learning framework for nonlinear partial differential equations. *Communications in Computational Physics*, 28(5), 2020.

[7] Mingqian Wang, Jianshan Zhou, Xuting Duan, Dezong Zhao, Pinlong Cai, Junda Zhai, Xinyu Liu, and Chenghao Ren. Nonlinear system identification for quadrotors with neural ordinary differential equations. In *2023 IEEE International Conference on Unmanned Systems (ICUS)*, pages 317–322. IEEE, 2023.

[8] Fangfei Cao and Jinkun Liu. Boundary control for pde flexible manipulators: Accommodation to both actuator faults and sensor faults. *Asian Journal of Control*, 24(4):1700–1712, 2022.

[9] Xingyu Zhou, Haoping Wang, Yang Tian, and Gang Zheng. Disturbance observer-based adaptive boundary iterative learning control for a rigid-flexible manipulator

with input backlash and endpoint constraint. *International Journal of Adaptive Control and Signal Processing*, 34(9):1220–1241, 2020.

[10] Wei He, Tingting Wang, Xiuyu He, Lung-Jieh Yang, and Okyay Kaynak. Dynamical modeling and boundary vibration control of a rigid-flexible wing system. *IEEE/ASME transactions on mechatronics*, 25(6):2711–2721, 2020.

[11] Yu Liu, Wenkang Zhan, Mali Xing, Yilin Wu, Ruifeng Xu, and Xinsheng Wu. Boundary control of a rotating and length-varying flexible robotic manipulator system. *IEEE Transactions on Systems, Man, and Cybernetics: Systems*, 52(1):377–386, 2020.

[12] Tingting Jiang, Jinkun Liu, and Wei He. Adaptive boundary control for a flexible manipulator with state constraints using a barrier lyapunov function. *Journal of dynamic systems, measurement, and control*, 140(8):081018, 2018.

[13] Haibin Dou and Shaoping Wang. A boundary control for motion synchronization of a two-manipulator system with a flexible beam. *Automatica*, 50(12):3088–3099, 2014.

[14] Wei He and Chen Sun. Boundary feedback stabilisation of a flexible robotic manipulator with constraint. *International Journal of Control*, 89(3):635–651, 2016.

[15] Ti Chen and Jinjun Shan. Distributed control of multiple flexible manipulators with unknown disturbances and dead-zone input. *IEEE Transactions on Industrial Electronics*, 67(11):9937–9947, 2019.

[16] Fareh Raouf, Saad Mohamad, Saad Maarouf, and Bettayeb Maamar. Distributed adaptive control strategy for flexible link manipulators. *Robotica*, 35(7):1562–1584, 2017.

[17] Owais Khan, Mahmood Pervaiz, Ejaz Ahmad, and Jamshed Iqbal. On the derivation of novel model and sophisticated control of flexible joint manipulator. *Revue Roumaine des Sciences Techniques-Serie Electrotechnique et Energetique*, 62(1):103–108, 2017.

[18] S Natsiavas and E Paraskevopoulos. A set of ordinary differential equations of motion for constrained mechanical systems. *Nonlinear Dynamics*, 79:1911–1938, 2015.

[19] Jan Awrejcewicz et al. Ordinary differential equations and mechanical systems. 2014.

[20] Benjamin C Koenig, Suyong Kim, and Sili Deng. Kan-odes: Kolmogorov–arnold network ordinary differential equations for learning dynamical systems and hidden physics. *Computer Methods in Applied Mechanics and Engineering*, 432:117397, 2024.

[21] Selcan Kaplanvural, Eren Tosyalı, and İsmail Ekmekçi. A new approach to determine occupational accident dynamics by using ordinary differential equations based on sir model. *Scientific Reports*, 14(1):23985, 2024.

[22] Zuoming Zou, Shuming Yang, and Liang Zhao. Dual-loop control and state prediction analysis of quav trajectory tracking based on biological swarm intelligent optimization algorithm. *Scientific Reports*, 14(1):19091, 2024.

[23] Yong Ren, Zhijia Zhao, Chunliang Zhang, Qinmin Yang, and Keum-Shik Hong. Adaptive neural-network boundary control for a flexible manipulator with input constraints and model uncertainties. *IEEE Transactions on Cybernetics*, 51(10):4796–4807, 2020.

[24] Yu Liu, Xiongbin Chen, Yanfang Mei, and Yilin Wu. Observer-based boundary control for an asymmetric output-constrained flexible robotic manipulator. *Science China. Information Sciences*, 65(3):139203, 2022.

[25] Zhijie Liu, Jinkun Liu, and Wei He. Partial differential equation boundary control of a flexible manipulator with input saturation. *International Journal of Systems Science*, 48(1):53–62, 2017.

[26] Zhijie Liu, Jinkun Liu, Zhijie Liu, and Jinkun Liu. Dynamic modeling and vibration control for a nonlinear three-dimensional flexible manipulator. *PDE Modeling and Boundary Control for Flexible Mechanical System*, pages 137–171, 2020.

[27] Sandra Hirche et al. Distributed control for cooperative manipulation with event-triggered communication. *IEEE Transactions on Robotics*, 36(4):1038–1052, 2020.

[28] Hui Ma, Qi Zhou, Hongyi Li, and Renquan Lu. Adaptive prescribed performance control of a flexible-joint robotic manipulator with dynamic uncertainties. *IEEE Transactions on Cybernetics*, 52(12):12905–12915, 2021.

[29] Kenneth L Hillsley and Stephen Yurkovich. Vibration control of a two-link flexible robot arm. *Dynamics and Control*, 3(3):261–280, 1993.

[30] Woosoon Yim, Jichun Zuang, and Sahjendra N Singh. Experimental two-axis vibration suppression and control of a flexible robot arm. *Journal of robotic systems*, 10(3):321–343, 1993.

[31] SK Tso, TW Yang, WL Xu, and ZQ Sun. Vibration control for a flexible-link robot arm with deflection feedback. *International journal of non-linear mechanics*, 38(1):51–62, 2003.

[32] Wenxi Zhang. Vibration avoidance method for flexible robotic arm manipulation. *Journal of the Franklin Institute*, 355(9):3968–3989, 2018.

[33] Jiantao Li and Hua Deng. Vibration suppression of rotating long flexible mechanical arms based on harmonic input signals. *Journal of Sound and Vibration*, 436:253–261, 2018.

[34] Deshan Meng, Weining Lu, Wenfu Xu, Yu She, Xueqian Wang, Bin Liang, and Bo Yuan. Vibration suppression control of free-floating space robots with flexible appendages for autonomous target capturing. *Acta Astronautica*, 151:904–918, 2018.

[35] Jin Wei, Dengqing Cao, Lun Liu, and Wenhu Huang. Global mode method for dynamic modeling of a flexible-link flexible-joint manipulator with tip mass. *Applied Mathematical Modelling*, 48:787–805, 2017.

[36] Ronald GKM Aarts and Jan B Jonker. Dynamic simulation of planar flexible link manipulators using adaptive modal integration. *Multibody System Dynamics*, 7:31–50, 2002.

[37] MO Tokhi, Z Mohamed, and AWI Hashim. Modelling of a flexible robot manipulator using finite element methods: A symbolic approach. *Journal of low frequency noise, vibration and active control*, 18(2):63–76, 1999.

[38] Martin Goubej, Jana Königsmarková, Ronald Kampinga, Jakko Nieuwenkamp, and Stéphane Paquay. Employing finite element analysis and robust control concepts in mechatronic system design-flexible manipulator case study. *Applied Sciences*, 11(8):3689, 2021.

[39] Tian Soon Lee and Esmail Ali Alandoli. A critical review of modelling methods for flexible and rigid link manipulators. *Journal of the Brazilian Society of Mechanical Sciences and Engineering*, 42:1–14, 2020.

[40] Prasenjit Sarkhel, Nilotpal Banerjee, and Nirmal Baran Hui. Fuzzy logic-based tuning of pid controller to control flexible manipulators. *SN Applied Sciences*, 2:1–11, 2020.

[41] Wei Zhao, Yu Liu, and Xiangqian Yao. Adaptive fuzzy containment and vibration control for multiple flexible manipulators with model uncertainties. *IEEE Transactions on Fuzzy Systems*, 31(4):1315–1326, 2022.

[42] Qingxin Meng, Xuzhi Lai, Ze Yan, Chun-Yi Su, and Min Wu. Motion planning and adaptive neural tracking control of an uncertain two-link rigid–flexible manipulator with vibration amplitude constraint. *IEEE Transactions on Neural Networks and Learning Systems*, 33(8):3814–3828, 2021.

[43] Dongyang Shang, Xiaopeng Li, Meng Yin, and Fanjie Li. Control method of flexible manipulator servo system based on a combination of rbf neural network and pole placement strategy. *Mathematics*, 9(8):896, 2021.

[44] Fengming Han and Yingmin Jia. Sliding mode boundary control for a planar two-link rigid-flexible manipulator with input disturbances. *International Journal of Control, Automation and Systems*, 18(2):351–362, 2020.

[45] Alessio Turetta, Giuseppe Casalino, and Andrea Sorbara. Distributed control architecture for self-reconfigurable manipulators. *The International Journal of Robotics Research*, 27(3-4):481–504, 2008.

[46] Mohamed B Trabia and Linda Z Shi. Design and tuning of a distributed fuzzy logic controller for flexible-link manipulators. *Journal of Intelligent & Fuzzy Systems*, 10(3-4):215–233, 2001.

The authors would like to express our sincere appreciation to the referee for his/her constructive yet critical comments and the efforts provided in helping us to improve the quality and presentation of the paper further.

Response to Reviewer No. 2:

Ms. No. PONE-D-24-52674

"Research on boundary control of vehicle-mounted flexible manipulator based on partial differential equations"

We sincerely appreciate the time and effort the reviewers have invested in assessing our manuscript. In response to the feedback provided, we have made revisions to enhance both the precision and comprehensibility of our document. To facilitate the reviewers' assessment of these changes, we have marked the sections modified in response to **Reviewer 2's comments** in **red**. We are grateful for the valuable insights that have significantly improved our manuscript.

1. **Comment 1:** *"The abstract delves heavily into technical details, such as mathematical derivations and control laws, without providing sufficient context or emphasizing the broader significance of the study for practical applications or the field as a whole."*

   Response:
   Thank you for your constructive comments on the abstract of our manuscript. We appreciate your insight regarding the overemphasis on technical details and the need for more context and broader significance.

   In response to your feedback, we have revised the abstract to provide more context about the importance and practical applications of vehicle-mounted flexible robotic arms (VFRAs) and to emphasize the broader significance of our findings within the field. We hope that these revisions will make the abstract more accessible and relevant to a wider audience, thereby highlighting the impact of our research.

   Modification

   ......

   Abstract

   Vehicle-mounted flexible robotic arms (VFRAs) play a pivotal role in enhancing operational capabilities in sectors where human intervention is limited due to accessibility issues or safety concerns, such as in hazardous environments or precision surgery. This paper introduces the latest generation of VFRAs that utilize advanced soft materials and are designed with elongated structures to provide greater flexibility and control. We present a novel mathematical model, derived using Hamilton's principle, which simplifies the analysis of the arm's dynamic behaviors by employing partial differential equations (PDEs). This model allows us to understand how these arms behave over time and space, classifying them as distributed parameter systems. Furthermore, we enhance the practical utility of these robotic arms by implementing a proportional-derivative (PD) based boundary control law to achieve precise control of movement and suppression of vibrations, which are critical for operations requiring high accuracy. The effectiveness and practical relevance of our approach are demonstrated through numerical simulations, confirming that

this advanced control strategy significantly improves the performance and reliability of VFRAs in real-world applications. These advancements not only pave the way for more sophisticated robotic implementations but also have broad implications for the future of automated systems in various industries.

Thank you once again for your guidance, which has helped us improve the clarity and impact of our manuscript.

2. **Comment 2:** *"While the abstract describes the proposed modeling and control strategies, it does not mention how these methods compare to existing approaches, leaving the reader unclear about the novelty or advantages of the work."*

   Response:
   Thank you for your insightful comments regarding the abstract of our manuscript. We appreciate your observations about the need for clarity on how our methods compare to existing approaches.

   Our paper primarily focuses on advancing the modeling and control of vehicle-mounted flexible robotic arms (VFRAs) using partial differential equations (PDEs) [5], [6], which allows for a more detailed and precise description of the dynamic characteristics of these systems than is possible with traditional ordinary differential equations (ODEs) [18], [19], [20], [21] and [22]. The use of boundary control methods [23], [24], [25], as opposed to distributed control strategies[26], [27], [28], is proposed for its simplicity and effectiveness in practical applications.

   While the emphasis of our work is on presenting these novel modeling and control strategies, we recognize the importance of contextualizing our approach within the broader field. To this end, we have clarified in our manuscript that our approach is distinct in its capability to provide finer granularity in the dynamic modeling of VFRAs. We have noted that while direct comparisons to other methods are not the focus of this paper, the advantages of our approach include increased accuracy and simplified control mechanisms, which are significant enhancements over traditional methods.

   We have revised the abstract to better highlight these points, ensuring that readers understand the novelty and benefits of our approach even in the absence of direct comparisons. We believe these revisions will make the value and contribution of our work clearer to the readership.

   Thank you once again for your constructive feedback, which has undeniably helped refine our presentation.

3. **Comment 3:** *"The introduction should clearly conclude with a distinct section highlighting the novel contributions of your work."*

   Response:

   Thank you for your valuable feedback. We acknowledge the importance of clearly articulating the novel contributions of our work in the introduction section. To address your comments, we have revised the passage to include a distinct conclusion that explicitly highlights the innovative aspects of our research on vibration control in vehicle-mounted flexible robotic arms (VFRAs).

Modification

......

Abstract

......

Vehicle-mounted flexible robotic arms (VFRAs) play a pivotal role in enhancing operational capabilities in sectors where human intervention is limited due to accessibility issues or safety concerns, such as in hazardous environments or precision surgery. This paper introduces the latest generation of VFRAs that utilize advanced soft materials and are designed with elongated structures to provide greater flexibility and control. We present a novel mathematical model, derived using Hamilton's principle, which simplifies the analysis of the arm's dynamic behaviors by employing partial differential equations (PDEs). This model allows us to understand how these arms behave over time and space, classifying them as distributed parameter systems. Furthermore, we enhance the practical utility of these robotic arms by implementing a proportional-derivative (PD) based boundary control law to achieve precise control of movement and suppression of vibrations, which are critical for operations requiring high accuracy. The effectiveness and practical relevance of our approach are demonstrated through numerical simulations, confirming that this advanced control strategy significantly improves the performance and reliability of VFRAs in real-world applications. These advancements not only pave the way for more sophisticated robotic implementations but also have broad implications for the future of automated systems in various industries.

......

Introduction

......

Based on our comprehensive study of model construction and PDE control methods for VFRAs, the novel contributions of our work include the development of an advanced control strategy to mitigate vibrations more effectively than existing methods. This approach not only improves operational safety and accuracy but also sets a new benchmark for future research in robotic arm applications. Here are the main outcomes of our research:

0.1 The introduction of a PDE-based modeling approach to simulate the dynamics of VFRAs represents a significant advancement over traditional methods that primarily use ODEs. This approach allows for a more precise modeling of spatially distributed parameters.

0.2 The development of a PD boundary control strategy based on Lyapunov stability theory is novel in its application to VFRAs, offering a systematic method to enhance vibration control which is critical for precise operations.

0.3 The extensive validation of our control strategies through simulation sets a new benchmark for the robustness and reliability of control systems in handling real-world disturbances in flexible robotic arms.

We believe that these contributions distinctly set our work apart from existing research and address critical challenges in the field of robotic control systems.

4. **Comment 4:** *" The literature review should benefit from more explorations of previous studies. This will provide a richer context and demonstrate how your work builds upon or diverges from established research."*

   Response:
   Thank you for your thoughtful feedback regarding the literature review section. We agree that a more comprehensive exploration of previous studies will provide a richer context and further highlight how our work builds upon or diverges from established research.

   In response to your suggestion, we have expanded the literature review to include additional relevant studies. Specifically, we have incorporated a discussion of recent advancements in boundary control, distributed control, and intelligent control methods for flexible robotic systems.

   Modification

   Introduction

   ......

   Due to their unique design, these flexible robotic arms are generally soft, allowing them to operate flexibly in narrow or confined spaces. This design often incorporates advanced soft materials or flexible alloys to better adapt to irregular shapes and surfaces. However, the flexible design of these VFRAs also leads to vibration issues, which are more common than in rigid robotic arms. These vibrations can affect the precision of the arm's operations, exacerbate mechanical fatigue, and even lead to operational errors [29], [30], [31] and [32]. Recognizing this challenge, our research focuses on developing innovative vibration control strategies for VFRAs. Through effective vibration suppression measures, we aim to significantly enhance the performance and reliability of the robotic arm, extend its lifespan, and expand its application potential in various complex environments [33], [34].

   ......

   Hang et al. investigated the effects of input saturation, inertia uncertainty, and actuator faults on flexible satellites and proposed an adaptive fault-tolerant control strategy based on a disturbance observer and a fault diagnosis observer . This strategy estimates and suppresses vibration disturbances caused by flexible appendages and actuator faults. Simulation results demonstrated that the proposed method effectively enhances the fault-tolerance performance and control precision of the system.

   ......

   There are numerous research methods for controlling VFRAs modeled by PDEs, such as boundary control [23], [24], [25], modal methods [35], [36], finite element methods [37], [38], [39], distributed control [26], [27], [28], and intelligent control methods like fuzzy logic [40], [41] and neural network control [42], [43]. While each method has its suitable applications and limitations—for instance, boundary control is advantageous for directly manipulating

the system's boundary conditions to effectively control the state [44], its implementation may require precise measurement and control of boundary conditions, which can be technically complex. On the other hand, distributed control improves the system's resistance to local disturbances through multi-point control [45], [46]. Yet, it necessitates complex communication and coordination mechanisms to ensure effective synchronization between control nodes, potentially demanding substantial computational resources and high-speed communication networks to maintain real-time efficiency and effectiveness. In the study by Zhou [9], an observer-based adaptive boundary iterative learning control method was developed to handle the challenges posed by input backlash and external disturbances in a dual-link rigid-flexible manipulator. Extensive theoretical analysis and numerical simulations have demonstrated the efficacy of this approach in diminishing vibrations and enhancing precision in tracking performance. Similar to Reference [9], He et al. also utilized boundary control methods to manage a dual-link rigid-flexible wing system, enhancing aircraft maneuverability and adaptability through biomimetic principles. The control strategy developed by their team successfully mitigated wing vibrations and ensured precise angular positioning. The system's stability was confirmed using Lyapunov's method, and numerical simulations further validated the effectiveness of the controller [10]. In contrast to boundary controller designs [11], [12], [13], [14], Chen et al. crafted a distributed control system by combining multiple control techniques, effectively tackling the challenges of coordinating multiple flexible robotic arms in demanding settings and maintaining system stability. The effectiveness of this approach was also demonstrated through experimental validation [15]. In [16], Raouf successfully developed an adaptive distributed control strategy tailored for complex flexible-link robotic manipulators. This innovative approach not only optimized dynamic tracking performance but also effectively reduced system vibrations. Its global stability and practical applicability were rigorously validated through real-world implementation, demonstrating its robustness and efficiency in handling challenging control scenarios.

We appreciate your valuable input, which has helped us improve the clarity and depth of our manuscript.

5. **Comment 5:** *" The discussion section needs to be expanded to more thoroughly analyze the results."*

   Response:
   Thank you for your valuable feedback regarding the discussion section. We appreciate your observation that the discussion could benefit from a more thorough analysis of the results. In response to your suggestion, we will expand the discussion section to provide a deeper analysis of the results.

   ......

   Modification

   Simulation results show:

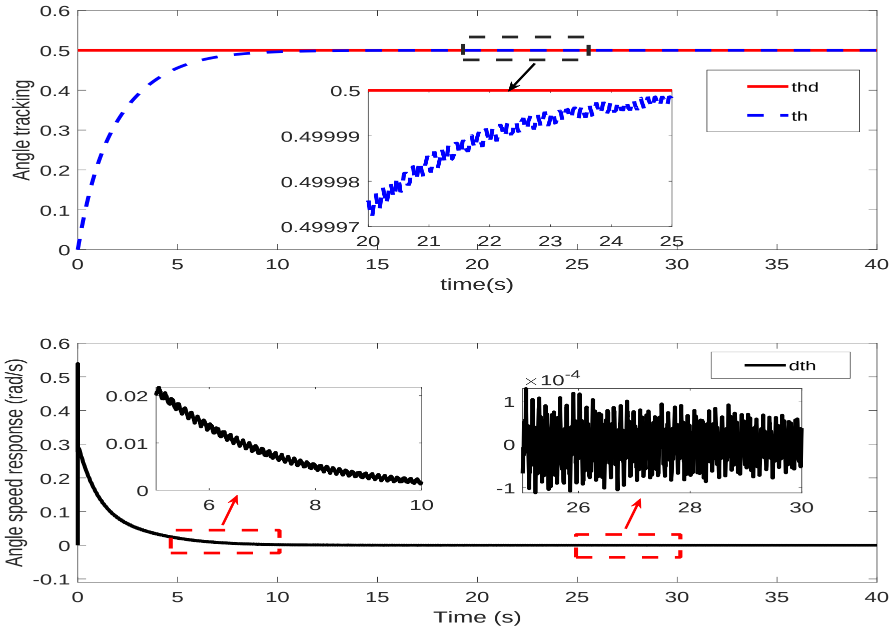

Figure 9: Angle and angular velocity response.

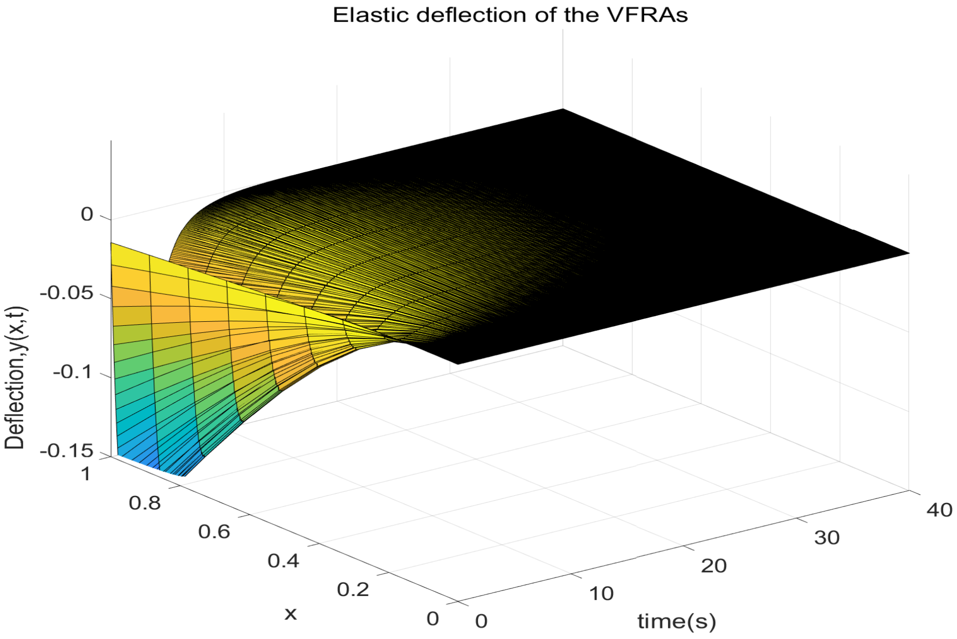

Figure 10: Three-dimensional graph of distributed elastic deformations on a VFRA.

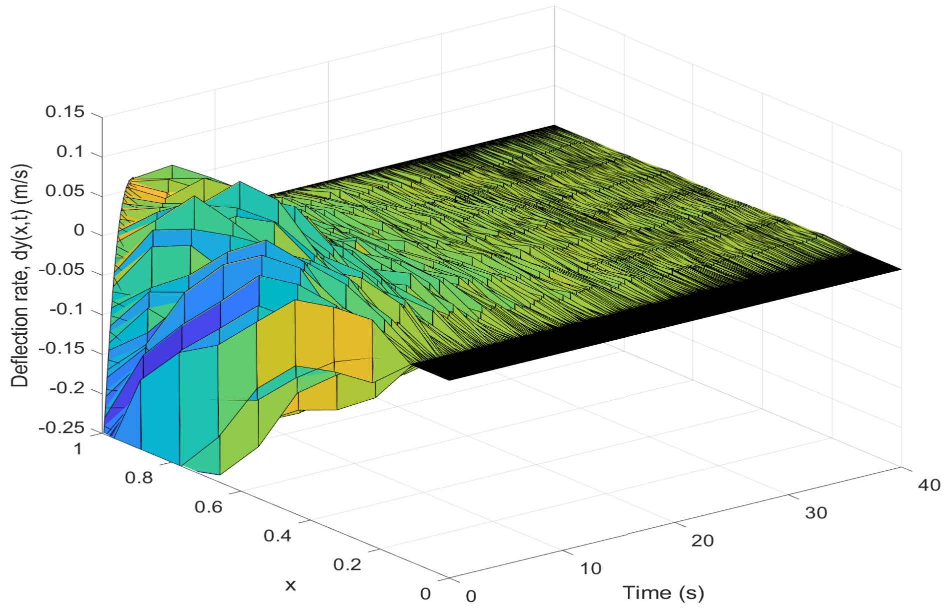

Figure 11: Three-dimensional graph of the rate of change in distributed elastic deformations on a VFRA.

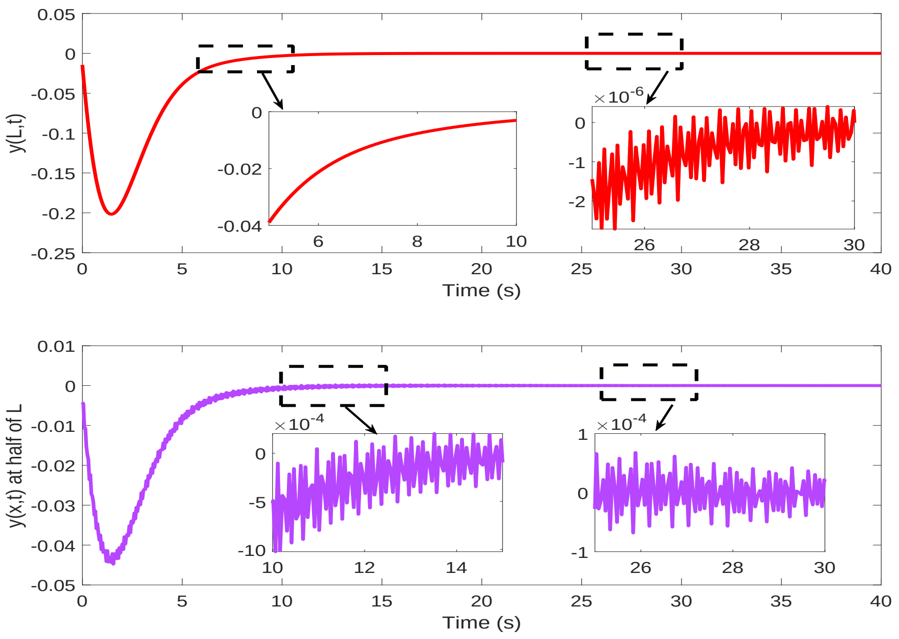

Figure 12: The schematic diagram of elastic deformation of the VFRA at $x = 0.5L$ and $x = L$.

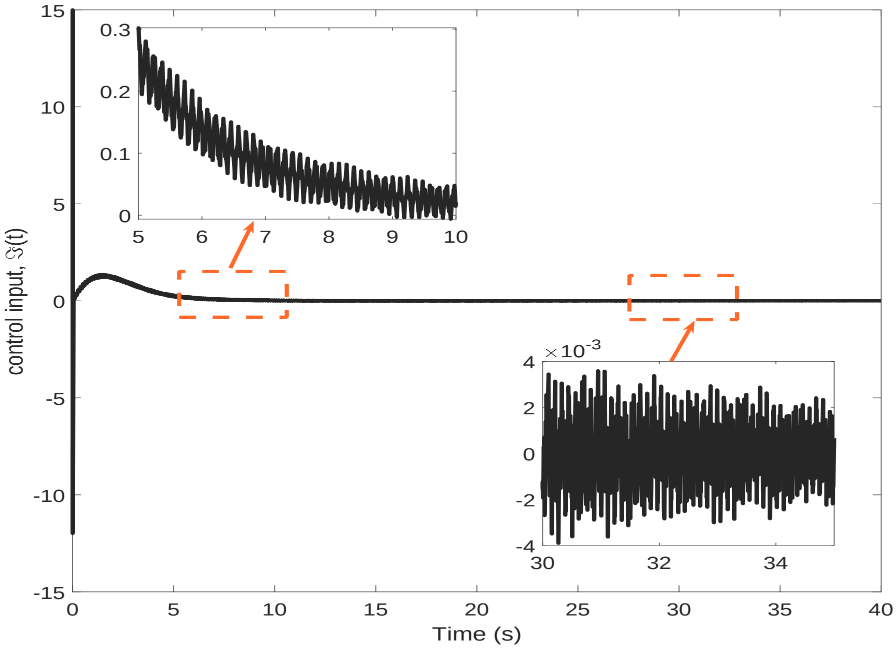

Figure 13: The control input $\Im(t)$.

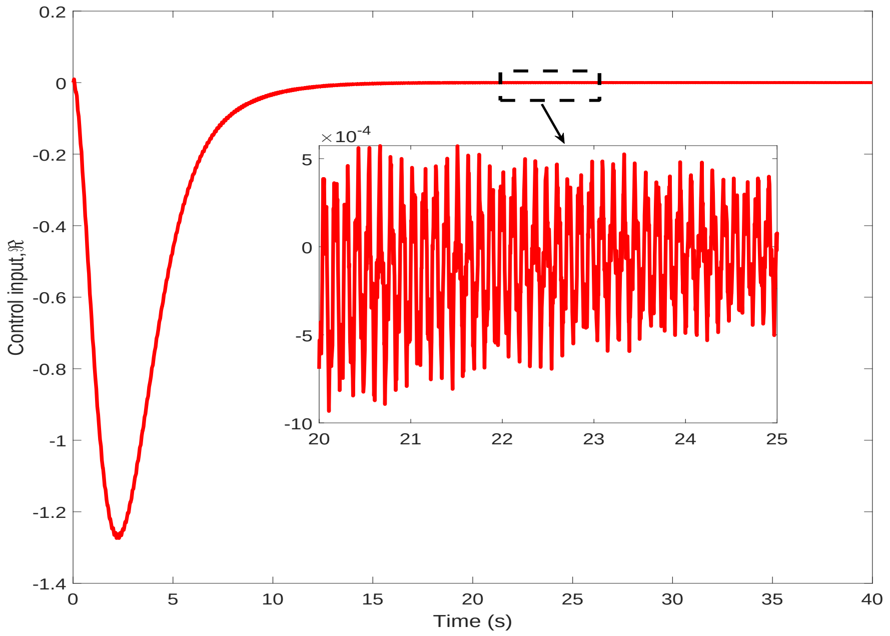

Figure 14: The control input $\Re(t)$.

....

Figure 9 displays the data graphs for angle and angular velocity responses. In the top graph of Figure 9, the red solid line (labeled ”thd”) represents the target angle, which is the ideal or desired angle set by the system. The blue dashed line (labeled ”-th”) shows the actual angle response. Initially, there is a noticeable discrepancy between the actual angle and the target angle as depicted by the blue and red lines not coinciding. However, as the control system adjusts, the actual angle gradually converges to the target, ultimately stabilizing close to the desired setting. This behavior underscores the effectiveness of the control strategy in correcting deviations over time, although the initial response time and overshoot could be areas for further improvement.

The bottom graph of Figure 9 illustrates how the angular velocity (i.e., the rate of change of angle) varies over time. The angular velocity initially exhibits a rapid decline from a high positive value to near zero, transitions through a negative phase, and finally stabilizes near zero. This portion of the graph, particularly the zoomed-in view between 26 and 30 seconds, highlights the fluctuations in angular velocity before stabilization, suggesting a critical examination of damping characteristics and response time of the control system to dynamic changes.

Figure 10 showcases the progression of elastic deformation in VFRAs at various time intervals. This plot vividly illustrates the temporal evolution of deformation, where minimal initial deformations intensify notably over time, with pronounced changes at specific positions along the x-axis. Such information is critical as it highlights how external forces dynamically influence the structural integrity of the manipulator. Conversely, Figure 11 presents a three-dimensional visualization of the deformation rate in VFRAs, offering insights into how quickly the material responds to applied forces. The slow initial deformation rate that sharply accelerates at certain positions along the x-axis can inform the design of more resilient flexible manipulators.

Figure 12 presents a comparative analysis of the elastic deformation of VFRAs at two key positions, x = 0.5L and x = L, where L is the total length of the VFRAs. The stark contrast in deformation patterns between these two points is evident; deformation at the midpoint (0.5L) is significantly greater and more intense than at the endpoint (L). This observation could have substantial implications for the design and utilization of VFRAs, indicating potential areas of vulnerability and the need for targeted reinforcement or control adjustments. Figure 13 and Figure 14 provide detailed visualizations of the control system’s input signals over time, emphasizing the system’s responsiveness to disturbances and its ability to maintain stability under dynamic conditions. These graphs are instrumental in understanding the oscillatory behavior and stability of the control system post-adjustment, serving as a foundation for further optimization of the control strategy.

6. **Comment 6:** *“ The first paragraph of the conclusion should succinctly summarize the contributions of the study in past tense, clearly stating what has been accomplished and the impact it has on the field.”*

   Response:

   Thank you for your insightful feedback regarding the conclusion section. We agree that the first paragraph should succinctly summarize the contributions of the study in the past

tense, clearly outlining what has been accomplished and its impact on the field. In response to your suggestion, we have revised the first paragraph of the conclusion to better align with these requirements.

Modification

Discussion and future work:

This study developed a dynamic modeling framework and control strategy for VFRAs using PDEs. By applying Hamilton's principle, we constructed a dynamic model that directly derived the system's governing PDEs, simplifying the analysis of complex forces acting on the arm. Additionally, a PD boundary control strategy was designed to achieve precise angle tracking and vibration suppression along the arm. The control approach was validated through numerical simulations, demonstrating its effectiveness in ensuring stability, enhancing control precision, and suppressing vibrations. These contributions provide a robust foundation for advancing the modeling and control of flexible robotic systems, improving their applicability in complex and dynamic environments.

We appreciate your guidance, which has significantly enhanced the clarity and impact of our manuscript.

7. **Comment 7:** *" The second paragraph of the conclusion should provide clear and actionable future recommendations."*

   Response:
   Thank you for your valuable feedback regarding the second paragraph of the conclusion. We appreciate your suggestion to provide clear and actionable future recommendations. In response, we have revised the paragraph to outline specific and focused directions for future research, highlighting actionable steps and their potential impact on the development of VFRAs.

   Modification

   Discussion and future work:

   Future research will focus on enhancing the real-time responsiveness of the dynamic model by incorporating advanced computational techniques such as machine learning and data-driven approaches. To improve the adaptability of the control strategy, we plan to design and test adaptive algorithms capable of handling varying environmental conditions and task requirements. Furthermore, reducing control input delays and mitigating uncertainties will be addressed by integrating predictive control frameworks and optimization-based algorithms, aiming to enhance system stability and reliability. These efforts will not only expand the application scope of VFRAs in complex operational environments but also ensure their performance and efficiency under increasingly demanding tasks.

   We believe these modifications enhance the clarity and relevance of the future recommendations section. Thank you once again for your thoughtful guidance.

8. **Comment 8:** *" Discussion should be expanded, future work is with conclusion in a new section."*

   Response:
   Thank you for your valuable feedback regarding the discussion and conclusion sections.

We appreciate your suggestion to expand the discussion and separate the future work into a new section.

Modification

5 Discussion and future work:

This study developed a dynamic modeling framework and control strategy for VFRAs using PDEs. By applying Hamilton's principle, we constructed a dynamic model that directly derived the system's governing PDEs, simplifying the analysis of complex forces acting on the arm. Additionally, a PD boundary control strategy was designed to achieve precise angle tracking and vibration suppression along the arm. The control approach was validated through numerical simulations, demonstrating its effectiveness in ensuring stability, enhancing control precision, and suppressing vibrations. These contributions provide a robust foundation for advancing the modeling and control of flexible robotic systems, improving their applicability in complex and dynamic environments.

Future research will focus on enhancing the real-time responsiveness of the dynamic model by incorporating advanced computational techniques such as machine learning and data-driven approaches. To improve the adaptability of the control strategy, we plan to design and test adaptive algorithms capable of handling varying environmental conditions and task requirements. Furthermore, reducing control input delays and mitigating uncertainties will be addressed by integrating predictive control frameworks and optimization-based algorithms, aiming to enhance system stability and reliability. These efforts will not only expand the application scope of VFRAs in complex operational environments but also ensure their performance and efficiency under increasingly demanding tasks.

We are confident that these revisions will enhance the clarity, depth, and overall quality of the manuscript. Thank you again for your insightful comments, which have been invaluable in improving our work.

References

[1] Changle Li, Quyuan Luo, Guoqiang Mao, Min Sheng, and Jiandong Li. Vehicle-mounted base station for connected and autonomous vehicles: Opportunities and challenges. *IEEE Wireless Communications*, 26(4):30–36, 2019.

[2] Yong Cheng, Jing Lu, and Can Wang. Design of a multiple band vehicle-mounted antenna. *International Journal of Antennas and Propagation*, 2019(1):6098014, 2019.

[3] Reza Sabzevari and Davide Scaramuzza. Multi-body motion estimation from monocular vehicle-mounted cameras. *IEEE Transactions on Robotics*, 32(3):638–651, 2016.

[4] Raza Ul Islam, Jamshed Iqbal, Sarah Manzoor, Aayman Khalid, and Sana Khan. An autonomous image-guided robotic system simulating industrial applications. In *2012 7th International Conference on system of systems engineering (SoSE)*, pages 344–349. IEEE, 2012.

[5] Steven L Brunton and J Nathan Kutz. Promising directions of machine learning for partial differential equations. *Nature Computational Science*, 4(7):483–494, 2024.

[6] Ameya D Jagtap and George Em Karniadakis. Extended physics-informed neural networks (xpinns): A generalized space-time domain decomposition based deep learning framework for nonlinear partial differential equations. *Communications in Computational Physics*, 28(5), 2020.

[7] Mingqian Wang, Jianshan Zhou, Xuting Duan, Dezong Zhao, Pinlong Cai, Junda Zhai, Xinyu Liu, and Chenghao Ren. Nonlinear system identification for quadrotors with neural ordinary differential equations. In *2023 IEEE International Conference on Unmanned Systems (ICUS)*, pages 317–322. IEEE, 2023.

[8] Fangfei Cao and Jinkun Liu. Boundary control for pde flexible manipulators: Accommodation to both actuator faults and sensor faults. *Asian Journal of Control*, 24(4):1700–1712, 2022.

[9] Xingyu Zhou, Haoping Wang, Yang Tian, and Gang Zheng. Disturbance observer-based adaptive boundary iterative learning control for a rigid-flexible manipulator with input backlash and endpoint constraint. *International Journal of Adaptive Control and Signal Processing*, 34(9):1220–1241, 2020.

[10] Wei He, Tingting Wang, Xiuyu He, Lung-Jieh Yang, and Okyay Kaynak. Dynamical modeling and boundary vibration control of a rigid-flexible wing system. *IEEE/ASME transactions on mechatronics*, 25(6):2711–2721, 2020.

[11] Yu Liu, Wenkang Zhan, Mali Xing, Yilin Wu, Ruifeng Xu, and Xinsheng Wu. Boundary control of a rotating and length-varying flexible robotic manipulator system. *IEEE Transactions on Systems, Man, and Cybernetics: Systems*, 52(1):377–386, 2020.

[12] Tingting Jiang, Jinkun Liu, and Wei He. Adaptive boundary control for a flexible manipulator with state constraints using a barrier lyapunov function. *Journal of dynamic systems, measurement, and control*, 140(8):081018, 2018.

[13] Haibin Dou and Shaoping Wang. A boundary control for motion synchronization of a two-manipulator system with a flexible beam. *Automatica*, 50(12):3088–3099, 2014.

[14] Wei He and Chen Sun. Boundary feedback stabilisation of a flexible robotic manipulator with constraint. *International Journal of Control*, 89(3):635–651, 2016.

[15] Ti Chen and Jinjun Shan. Distributed control of multiple flexible manipulators with unknown disturbances and dead-zone input. *IEEE Transactions on Industrial Electronics*, 67(11):9937–9947, 2019.

[16] Fareh Raouf, Saad Mohamad, Saad Maarouf, and Bettayeb Maamar. Distributed adaptive control strategy for flexible link manipulators. *Robotica*, 35(7):1562–1584, 2017.

[17] Owais Khan, Mahmood Pervaiz, Ejaz Ahmad, and Jamshed Iqbal. On the derivation of novel model and sophisticated control of flexible joint manipulator. *Revue Roumaine des Sciences Techniques-Serie Electrotechnique et Energetique*, 62(1):103–108, 2017.

[18] S Natsiavas and E Paraskevopoulos. A set of ordinary differential equations of motion for constrained mechanical systems. *Nonlinear Dynamics*, 79:1911–1938, 2015.

[19] Jan Awrejcewicz et al. Ordinary differential equations and mechanical systems. 2014.

[20] Benjamin C Koenig, Suyong Kim, and Sili Deng. Kan-odes: Kolmogorov–arnold network ordinary differential equations for learning dynamical systems and hidden physics. *Computer Methods in Applied Mechanics and Engineering*, 432:117397, 2024.

[21] Selcan Kaplanvural, Eren Tosyalı, and İsmail Ekmekçi. A new approach to determine occupational accident dynamics by using ordinary differential equations based on sir model. *Scientific Reports*, 14(1):23985, 2024.

[22] Zuoming Zou, Shuming Yang, and Liang Zhao. Dual-loop control and state prediction analysis of quav trajectory tracking based on biological swarm intelligent optimization algorithm. *Scientific Reports*, 14(1):19091, 2024.

[23] Yong Ren, Zhijia Zhao, Chunliang Zhang, Qinmin Yang, and Keum-Shik Hong. Adaptive neural-network boundary control for a flexible manipulator with input constraints and model uncertainties. *IEEE Transactions on Cybernetics*, 51(10):4796–4807, 2020.

[24] Yu Liu, Xiongbin Chen, Yanfang Mei, and Yilin Wu. Observer-based boundary control for an asymmetric output-constrained flexible robotic manipulator. *Science China. Information Sciences*, 65(3):139203, 2022.

[25] Zhijie Liu, Jinkun Liu, and Wei He. Partial differential equation boundary control of a flexible manipulator with input saturation. *International Journal of Systems Science*, 48(1):53–62, 2017.

[26] Zhijie Liu, Jinkun Liu, Zhijie Liu, and Jinkun Liu. Dynamic modeling and vibration control for a nonlinear three-dimensional flexible manipulator. *PDE Modeling and Boundary Control for Flexible Mechanical System*, pages 137–171, 2020.

[27] Sandra Hirche et al. Distributed control for cooperative manipulation with event-triggered communication. *IEEE Transactions on Robotics*, 36(4):1038–1052, 2020.

[28] Hui Ma, Qi Zhou, Hongyi Li, and Renquan Lu. Adaptive prescribed performance control of a flexible-joint robotic manipulator with dynamic uncertainties. *IEEE Transactions on Cybernetics*, 52(12):12905–12915, 2021.

[29] Kenneth L Hillsley and Stephen Yurkovich. Vibration control of a two-link flexible robot arm. *Dynamics and Control*, 3(3):261–280, 1993.

[30] Woosoon Yim, Jichun Zuang, and Sahjendra N Singh. Experimental two-axis vibration suppression and control of a flexible robot arm. *Journal of robotic systems*, 10(3):321–343, 1993.

[31] SK Tso, TW Yang, WL Xu, and ZQ Sun. Vibration control for a flexible-link robot arm with deflection feedback. *International journal of non-linear mechanics*, 38(1):51–62, 2003.

[32] Wenxi Zhang. Vibration avoidance method for flexible robotic arm manipulation. *Journal of the Franklin Institute*, 355(9):3968–3989, 2018.

[33] Jiantao Li and Hua Deng. Vibration suppression of rotating long flexible mechanical arms based on harmonic input signals. *Journal of Sound and Vibration*, 436:253–261, 2018.

[34] Deshan Meng, Weining Lu, Wenfu Xu, Yu She, Xueqian Wang, Bin Liang, and Bo Yuan. Vibration suppression control of free-floating space robots with flexible appendages for autonomous target capturing. *Acta Astronautica*, 151:904–918, 2018.

[35] Jin Wei, Dengqing Cao, Lun Liu, and Wenhu Huang. Global mode method for dynamic modeling of a flexible-link flexible-joint manipulator with tip mass. *Applied Mathematical Modelling*, 48:787–805, 2017.

[36] Ronald GKM Aarts and Jan B Jonker. Dynamic simulation of planar flexible link manipulators using adaptive modal integration. *Multibody System Dynamics*, 7:31–50, 2002.

[37] MO Tokhi, Z Mohamed, and AWI Hashim. Modelling of a flexible robot manipulator using finite element methods: A symbolic approach. *Journal of low frequency noise, vibration and active control*, 18(2):63–76, 1999.

[38] Martin Goubej, Jana Königsmarková, Ronald Kampinga, Jakko Nieuwenkamp, and Stéphane Paquay. Employing finite element analysis and robust control concepts in mechatronic system design-flexible manipulator case study. *Applied Sciences*, 11(8):3689, 2021.

[39] Tian Soon Lee and Esmail Ali Alandoli. A critical review of modelling methods for flexible and rigid link manipulators. *Journal of the Brazilian Society of Mechanical Sciences and Engineering*, 42:1–14, 2020.

[40] Prasenjit Sarkhel, Nilotpal Banerjee, and Nirmal Baran Hui. Fuzzy logic-based tuning of pid controller to control flexible manipulators. *SN Applied Sciences*, 2:1–11, 2020.

[41] Wei Zhao, Yu Liu, and Xiangqian Yao. Adaptive fuzzy containment and vibration control for multiple flexible manipulators with model uncertainties. *IEEE Transactions on Fuzzy Systems*, 31(4):1315–1326, 2022.

[42] Qingxin Meng, Xuzhi Lai, Ze Yan, Chun-Yi Su, and Min Wu. Motion planning and adaptive neural tracking control of an uncertain two-link rigid–flexible manipulator with vibration amplitude constraint. *IEEE Transactions on Neural Networks and Learning Systems*, 33(8):3814–3828, 2021.

[43] Dongyang Shang, Xiaopeng Li, Meng Yin, and Fanjie Li. Control method of flexible manipulator servo system based on a combination of rbf neural network and pole placement strategy. *Mathematics*, 9(8):896, 2021.

[44] Fengming Han and Yingmin Jia. Sliding mode boundary control for a planar two-link rigid-flexible manipulator with input disturbances. *International Journal of Control, Automation and Systems*, 18(2):351–362, 2020.

[45] Alessio Turetta, Giuseppe Casalino, and Andrea Sorbara. Distributed control architecture for self-reconfigurable manipulators. *The International Journal of Robotics Research*, 27(3-4):481–504, 2008.

[46] Mohamed B Trabia and Linda Z Shi. Design and tuning of a distributed fuzzy logic controller for flexible-link manipulators. *Journal of Intelligent & Fuzzy Systems*, 10(3-4):215–233, 2001.

The authors would like to extend our heartfelt gratitude to the reviewer for their thorough and insightful comments, which have been instrumental in enhancing the quality and clarity of our manuscript. We deeply value the time and effort they have dedicated to providing constructive feedback and thoughtful suggestions, which have helped us to refine the content and presentation of our work further.
